# Seaweed Fly Larvae Cultivated on Macroalgae Side Streams: A Novel Marine Protein and Omega-3 Source for Rainbow Trout

**DOI:** 10.1155/2024/4221883

**Published:** 2024-10-07

**Authors:** Niklas Warwas, Emma L. Berdan, Xintian Xie, Elisabeth Jönsson, Jonathan A. C. Roques, Darragh Doyle, Markus Langeland, James Hinchcliffe, Henrik Pavia, Kristina Sundell

**Affiliations:** ^1^Department of Biological and Environmental Sciences, University of Gothenburg, Gothenburg, Sweden; ^2^Swedish Mariculture Research Center, SWEMARC, University of Gothenburg, Gothenburg, Sweden; ^3^Blue Food, Center for Future Seafood, University of Gothenburg, Gothenburg, Sweden; ^4^Department of Marine Sciences, University of Gothenburg, Tjärnö, Gothenburg, Sweden; ^5^RISE Research Institute of Sweden, Gothenburg, Sweden

**Keywords:** alternative feed ingredients, black soldier fly, intestinal health, marine insects, omega n-3 fatty acids, salmonid nutrition

## Abstract

A nutritional bottleneck in salmonid aquaculture is the procurement of marine-derived compounds, such as essential amino and fatty acids, including omega-3 fatty acids, lysine, and methionine. Therefore, insects containing these compounds are highly promising as feed ingredients. The present study evaluates larvae of a “marine” insect (*Coelopa frigida*, the bristly-legged seaweed fly larvae, SWFL) reared on brown algae side streams as a feed ingredient for rainbow trout (*Oncorhynchus mykiss*). SWFL contained, on a dry matter basis, 60% crude protein (CP), 3.5% lysine, and 1.5% methionine, as well as 17% lipids, including 4% eicosapentaenoic acid and docosahexaenoic acid. Four isoenergetic (*ca*. 23 MJ kg^−1^ gross energy) and isoproteic (*ca*. 45% CP) feeds were evaluated in a 10-week feeding trial. The diets included a control containing 25% fish meal, a commercial reference, and two diets substituting 40% fish meal with either SWFL or partially defatted black soldier fly larvae (BSFL) meal (*Hermetia illucens*). SWFL-fed fish displayed higher specific growth rates (SGR) compared to BSFL-fed fish and similar SGR compared to control and reference diet-fed fish. Feed intake in SWFL-fed fish was higher than for the control and BSFL diets and similar to the reference diet. The feed conversion ratio of fish fed the BSFL and SWFL diets was higher compared to the control, suggesting lower bioavailability of both insect meals compared to fish meals. No difference in intestinal health parameters was noted between the insect diets and the control diet, indicating good intestinal health across all treatments. However, changes in electrogenic intestinal transport were observed between the SWFL and BSFL diets, illustrating the heterogeneous effect of different insect products. Overall, SWFL meal is a promising alternative marine feed ingredient, compatible with circular production systems, as it can be efficiently cultivated using marine side streams.

## 1. Introduction

While capture fisheries have stagnated for decades, the aquaculture sector has expanded rapidly to meet the growing demand for seafood [1]. As a result, finfish aquaculture currently accounts for roughly 50% of all fish consumed globally [1]. With about 2 million metric tons per year, the farming of salmonid fish accounts for the largest part of European aquaculture [2, 3]. The rapid expansion of salmonid aquaculture is attributable to many factors including high demand, technological advancements, improved husbandry, and optimized nutrition [4, 5]. While the sector continues to evolve, one of the major challenges for the future sustainable development of finfish aquaculture is the continued supply of sustainably sourced fish feed [6, 7]. Furthermore, feed accounts for over 50% of the climate footprint of finfish aquaculture [83]. Therefore, sustainable aquaculture will only be achievable with the development of more sustainable feed.

Fed aquaculture is largely dependent on high-quality feed ingredients, which are intended to ensure optimal health and growth of the animal [8]. Fish meal is considered the “golden standard” protein source due to its high digestibility, high palatability, and well-balanced nutrient profile, especially for carnivorous marine fish [9]. However, the supply of fish (meal) from pelagic capture fisheries has been stagnating for decades while the need for sustainable raw materials in feeds continues to increase [1, 10]. Today, plant-based ingredients account for the biggest share of modern compound feeds due to advantages in price and availability. Nonetheless, plant ingredients (such as legumes, oil seeds, and grains) demand limited resources, such as arable land and freshwater, may compete with direct human consumption, and display less optimal nutritional profiles compared to fish meal [11, 12]. Additionally, plant-based ingredients, as well as algae, may contain high amounts of fiber and carbohydrates (up to 60%) and include antinutrients, such as tannins, saponins, and protease inhibitors [13–15]. Such compounds may reduce feed intake (FI) and digestibility and can induce intestinal inflammation, other pathological states, and stress [16, 17]. As the primary interface between feed and organism, an assessment of intestinal health is therefore crucial when evaluating novel feed ingredients.

In recent years, there has been a surge in the development of insects as a protein source in fish feed, the most popular being the black soldier fly larvae (BSFL; *Hermetia illucens*). BSFL can efficiently convert a wide variety of low-value organic matter into larval biomass [18, 19]. The nutrient content of BSFL is influenced by rearing substrate and harvest time, but they generally contain around 30% lipids and 40% protein on a dry matter (DM) basis, as well as a favorable amino acid profile [20–23]. Insects are also part of the natural diet of rainbow trout (*Oncorhynchus mykiss*), and juvenile Atlantic salmon (*Salmo salar*), and several studies have demonstrated high acceptability and bioavailability of BSFL in diets for salmonid fish [13, 24, 25]. However, previous studies have also identified negative effects on feed conversion, digestibility, and growth when including high levels of BSFL meal (>20%), especially when substituting fish meal [26–28]. Furthermore, BSFL contain high levels of short-chain fatty acids, which are less desirable for salmonid fish which require long-chained polyunsaturated fatty acids (LC-PUFAs), especially docosahexaenoic acid (DHA) and eicosapentaenoic acid (EPA; [20, 21, 29]). These LC-PUFAs are virtually absent in BSFL when cultured on terrestrial side streams; therefore, efforts have been made to cultivate BSFL on marine substrates (e.g., fish processing sides streams and algae) to increase their omega-3 LC-PUFA content [19, 21, 30–33]. Utilizing seaweeds is especially promising as they contain high levels of LC-PUFAs but also high levels of carbohydrates and moderate-to-low levels of protein, limiting their direct inclusion in salmonid diets. At the same time, seaweed aquaculture is growing fast with annual growth rates of 6%–8% per year, accounting today for an annual production of *ca*. 35 million tonnes, resulting in increasing amounts of side streams [1, 34]. However, terrestrial insects such as BSFL appear to grow less efficiently on marine substrates, exhibiting both lower protein content and longer life cycles [21, 32, 35]. Therefore, BSFL may not be the optimal vector to valorize marine side streams, while insect species that naturally utilize marine side streams for growth may be more suitable for use in salmonid feed production.

So-called marine or intertidal insects are likely candidates, as many of these species naturally consume shore-washed algae and their associated bacterial biomass [36]. Larvae of the bristly legged seaweed fly, *Coelopa frigida*, stand out as particularly promising for several reasons. First, *C. frigida* larvae can grow efficiently on marine algae [37]. Second, they display a promising nutrient profile, including high levels of omega-3 fatty acids, while maintaining protein contents above 50% [37]. Despite these promising characteristics, the suitability of *C. frigida* as an ingredient in fish feed remains unassessed.

The aim of this study was to evaluate the potential of *C. frigida* larvae as a feed ingredient for salmonid fish. The first objective of the study was to produce *C. frigida* using locally sourced brown algal side streams and to carry out detailed chemical analyses to determine the nutrient composition of *C. frigida* larvae. The second objective was to carry out a feeding trial assessing the effect of *C. frigida* larvae meal compared to BSFL meal and fish meal on appetite, growth physiology, intestinal health, and nutrient utilization in rainbow trout.

## 2. Materials and Methods

### 2.1. *C. frigida* Cultivation

The seaweed fly larvae (SWFL) used in this study were reared using one part beach-cast bladderwrack (*Fucus vesiculosus*) from the Tjärnö archipelago on the Swedish west-coast and three parts farmed sugar kelp (*Saccharina latissima*), supplied by Nordic Seafarm (Nordic SeaFarm AB, Gothenburg, Sweden). Adult flies were introduced to the culture to lay eggs. After hatching, the larvae were cultured at high humidity (>90%) and at a temperature of 25°C for a growth period of 7 days. The SWFL were then harvested by hand (size *ca*. 40–50 mg) and stored untreated at −80°C until the larval meal production. To produce the meal, SWFL were thawed at room temperature, and second and third-instar stage larvae were separated from pupae and cleared from decomposed feeding substrate using tweezers. The cleaned larvae were dried in an oven at 50°C overnight and ground to a fine powder using a coffee grinder (Andersson Coffee Grinder CEG 1.0, Borås, Sweden). The SWFL meal (*ca*. 2.5 kg) was stored at −80°C before it was sent to the Feed Technology Laboratory (FTL), Swedish University of Agricultural Sciences (SLU), Uppsala, Sweden for feed production. Partially defatted BSFL meal was purchased from Protix Biosystems BV (Dongen, Netherlands), which is commonly used in commercial aquafeeds. Instead of a full-fat BSFL meal, a defatted meal was chosen as it displayed a higher protein content (*ca*. 60% as opposed to 40%) and therefore resembled the SWFL meal more closely.

### 2.2. Feed Formulation and Production

Three experimental diets were formulated to be isoenergetic (*ca*. 23 MJ kg^−1^ gross energy), isoproteic (*ca*. 45% crude protein [CP]), and isolipidic (*ca*. 23% crude fat, Table 1). The control diet (C) contained 25% fish meal (low-temperature fish meal, North Atlantic origin), 16% soy protein concentrate, 13.4% wheat gluten, 14% wheat meal, and 9% fava bean meal as protein sources. The lipids in the diet were commercially obtained fish oil (10%) and rapeseed oil (9%). The two remaining experimental diets were formulated to substitute 40% of fish meal, on a protein basis, with either SWFL or BSFL meal and balanced using soy protein concentrate and fava bean meal to maintain isoenergetic, isoproteic, and isolipidic conditions. All three diets were extruded at the FTL using a twin-screw extruder (3-mm screen), and the oil fraction was added through vacuum coating. Directly after the production, the diets were shipped to the Department of Biological and Environmental Sciences (BioEnv) at the University of Gothenburg, Gothenburg, Sweden, and stored at 4°C until the start of the feeding trial. Furthermore, a commercial diet (Efico Enviro 921 Advance, BioMar AB Denmark, pellet size: 3 mm) was chosen as a reference (Ref) due to its comparable protein, lipid, and energy content levels.

### 2.3. Chemical Analysis

The composition of the experimental ingredients (SWEFL and BSFL), as well as the experimental diets, was analyzed as outlined by Warwas et al. [38]. For DM, the content was determined after drying the samples for 16 h at 103°C. The samples were then cooled in a desiccator and weighed. To determine the ash content, samples were burned at 550°C until the ash turned completely white [39]. The samples were then cooled as described above. The CP content was determined by multiplying the total nitrogen content by 6.25, according to the Nordic Committee on Feed Analysis [40]. The nitrogen content of the samples was analyzed according to Kjeldahl using a 2020 digester and a 2400 Kjeltec Analyser unit (FOSS Analytical A/S, Hillerød, Denmark). Determination of crude lipid (CL) content was carried out using hydrolyzation (1047 Hydrolysing Unit, Soxtec System HT 1043 Extraction Unit; FOSS Analytical A/S, Hillerød, Denmark). Neutral detergent fiber (NDF) was analyzed as per the procedure described by Mertens [41]. The gross energy content was analyzed using isoperibol bomb calorimetry (GE, Parr 6300, Parr Instrument Company, Moline, IL, USA). Amino and fatty acid contents were determined using high-performance liquid chromatography and gas chromatography (after conversion to fatty acid methyl esters), respectively. These analyses were carried out at Eurofins Food and Agro Testing Sweden AB (Linköping, Sweden).

### 2.4. Experimental Fish and Holding Conditions

The feeding trial was conducted in a freshwater recirculating aquaculture system (FW-RAS) at BioEnv between February and April 2021. Around 280 juvenile rainbow trout with an initial weight of *ca*. 30 g were purchased from Vänneåns Fiskodling AB (Knäred, Sweden). All fish were randomly distributed to seven cylindrical fiberglass tanks (around 40 fish per 100 L tank, flow rate *ca*. 2 L min^−1^) connected to the 20 m^3^ FW-RAS for acclimation to the system. The tanks were covered with semi-opaque plexiglass lids for partial shading. The photoperiod was set to 12 h light and dark, respectively. Water quality was monitored continuously for temperature (9.86 ± 0.18°C, mean ± SD [standard deviation]) and pH (7.28 ± 0.23) and three times per week for ammonia (NH_4_^+^ < 1 mg L⁻^1^), nitrite (NO_2_− < 0.5 mg L⁻^1^), nitrate (NO_3_− < 20 mg L⁻^1^), and dissolved oxygen (O_2_ > 8.0 mg L⁻^1^). During the acclimation period, fish were fed three times per week until satiation (Monday, Wednesday, and Friday) using commercial feed (1.1 mm, INICIO917, BioMar AB, Brande, Denmark).

After 3 weeks, all fish were anesthetized with tricaine methanesulfonate (MS-222, 80 mg L^−1^, Argent Chemical Laboratories, Redmond, WA, USA) and individually tagged using passive integrated transponders (PIT, Biomark, Boise, ID, USA). The PIT tags were implanted into the peritoneal cavity through a small lateral incision made between the pectoral and pelvic fins using a sterile scalpel. The weight (36.1 ± 7.5 g) and body length (14.3 ± 1.1 cm) of the individual fish were recorded. The feeding regime was changed to hand feeding twice per day (9:30 and 15:30) to habituate the fish to the experimental conditions. After 1 week of recovery, a total of 216 fish, with an average weight of 36.5 ± 5.9 g, were randomly redistributed into the 12 experimental tanks (18 fish per tank).

### 2.5. Feeding Trial

The experimental diets were randomly assigned to 12 experimental tanks (triplicate tanks/diet), and fish were fed twice daily, every day for 10 weeks. The initial daily feed ratio was set to 1.5% of initial body weight (IBW). Thereafter, it was increased in 10% increments (i.e., from 1.5% to 1.65% to 1.815%, etc.) weekly or after two consecutive days with feed waste of less than 5%. Uneaten pellets settled at the conical tank bottom and were collected using a net (0.5 mm) at the tank outlet 30 min after each feeding session by temporary flushing through lifting the standpipe. To determine the daily FI, the uneaten pellets were oven-dried overnight at 37°C and weighed. A pellet recovery test was carried out to validate the pellet collection and drying technique. For each diet, 100 g of the feed was submerged into water from the FW-RAS system for 1 h after which the pellets were collected and oven-dried as described above.

### 2.6. Blood and Tissue Sampling of Fish

The weight and length of each fish were measured at the start (day 0), middle (day 35), and end (day 70–72) of the trial. In the first two samplings, the fish were anesthetized with MS-222 (80 mg L^−1^). At the final sampling, the fish were euthanized using an overdose of Aquacalm (10 mg L^−1^, Metomidate hydrochloride, Syndel, Canada), followed by a sharp blow to the head.

At the final sampling, eight fish were netted randomly from each tank. Blood samples (1–2 mL) were taken from the caudal vessel using heparinized syringes. Hematocrit (Hct) and hemoglobin (Hb) were analyzed immediately. Aliquots of 80 μL were drawn into capillary tubes, centrifuged at 10,000 rcf for 5 min (Haematokrit 210, Hettich, Tuttlingen, Germany), and Hct was analyzed in duplicate using a Hawksley reader. Hb was analyzed using the Hb 201 + meter (Hemocue AB, Ängelholm, Sweden). All values were adjusted according to Clark et al. [42] to account for differences between human and fish blood. Using Hct and Hb, the mean corpuscular hemoglobin content (MCHC) was calculated (Equation (1)). The remaining blood was centrifuged at 10,000 rcf for 5 min (Thermo Scientific Heraeus Pico 17, Thermo Fisher Scientific, Waltham, MA, USA) for the separation of cells and plasma. Blood plasma samples were stored in 0.5 mL tubes at −80°C until analyses.

After blood samples were taken, the peritoneal cavity of the fish was opened laterally. The intestine, from the last pyloric ceca to the rectum, of four fish was excised using blunt dissection. The intestine was divided at the ileo-rectal valve into two regions, hereafter referred to as proximal and distal intestine. Each region was further divided into two sections, one for histological analysis and one for *ex vivo* Ussing chamber analysis. For histology, a 2-mm wide ring of the anterior-most part of each region was transferred to buffered formaldehyde (4%) for 24 h at 4°C and thereafter transferred to 70% ethanol. For *ex vivo* analysis using the Ussing chamber technique, a 2-cm intestinal segment was rinsed, placed in the ice-cold Ringer's solution (140 mM NaCl, 2.5 mM KCl, 15 mM NaHCO_3_, 1.5 mM CaCl_2_, 1 mM KH_2_PO_4_, 0.8 mM MgSO_4_, 10 mM glucose, 20 mM glutamine, 5 mM HEPES buffer and 0.5 mM lysine, pH 7.8) and transferred to an adjacent dry lab. The liver of the same four fish was removed and weighed for calculation of hepatosomatic index (HSI, Equation (2)). The hypothalamus was removed by decapitating the fish and laterally opening the cranium at the dorsal plain. The hypothalamus was separated from the midbrain and pituitary gland using fine tweezers and scalpel, wrapped in aluminum foil, snap-frozen in liquid nitrogen, and stored at −80°C until the gene expression analysis.

From the remaining four fish sampled, the viscera were weighed for calculation of the viscerosomatic index (VSI, Equation (3)). In addition, four fish from each tank were randomly selected, euthanized as described above, and stored at −80°C for whole-body composition analysis.

### 2.7. Ussing Chamber Analysis

The barrier and transport functions of the intestinal epithelium were assessed using the Ussing chamber methodology according to Sundell et al. [43] with modifications by Sundell and Sundh [44] as described by Warwas et al. [45]. The segments were opened longitudinally using blunt dissection, and a serosal layer of each intestinal segment was carefully removed under a stereo microscope using fine tweezers. This was performed to minimize diffusion barriers and ensure maximum oxygenation of the tissue. The peeled intestinal segments were mounted in the Ussing chamber (0.75 cm^2^ exposure area), and 4 mL of Ringer's solution was added to each half chamber. A gas mixture containing air and 0.3% CO_2_ was used to maintain a pH of 7.8 as well as to supply the tissue with oxygen. The temperature in the chambers was kept at 10°C using a water-supplied cooling mantle. To measure the electrochemical parameters of the epithelium, alternating adaptive DC voltages were applied to the epithelium, generating corresponding currents (*I*). The potential (*U*) across the epithelium was measured using Ag/AgCl electrodes. The respective *U*/*I* pairs were plotted, and a straight line was fitted using the least squares method to determine the transepithelial resistance (TER, slope), the short circuit current (SCC, *U* = 0), and the transepithelial potential (TEP, *I* = 0). TER represents epithelial barrier function and, for the intestinal epithelium of fish, reflects largely the paracellular shunt resistance through the tight junctions. TEP and SCC are largely generated by active transport processes of the epithelium, where TEP reflects the net total ion distribution generated by both active and passive processes, and SCC is the current generated by active transport processes across the epithelium.

During the first 60 min, the electrical parameters were allowed to stabilize after mounting of the tissue. After this acclimation time, the Ringer's solution was renewed, and 4.55 μL mL^−1 14^C-mannitol (1.2 × 1014 dpm mol^−1^; Moravek Biochemicals, Brea, CA, USA) and 0.45 μL mL^−1 3^H-Lysine (7.1 × 1016 dpm mol^−1^, Moravek Biochemicals Brea, CA, USA) were added to the mucosal half chamber to assess the apparent paracellular permeability (mannitol) and active amino acid uptake kinetics (lysine). Samples of 100 μL were taken from the serosal half chambers at minutes 0, 20, 25, 30, 60, 80, 85, and 90 and analyzed in a liquid scintillation counter using a dual label protocol (Wallac 1409, LKB Instruments, Turku, Finland) and 4 mL scintillation fluid (Ultima Gold, PerkinElmer, Waltham, USA).

### 2.8. Histology

The fixed intestinal samples were dehydrated using an ethanol gradient over 12 h and embedded in paraffin using a tissue processor (TP 1020, Leica, Wetzlar, Germany). From each intestinal section, six nonconsecutive 5 μm thick cross-sections were produced using a microtome (Shandon Scientific; Labex Instrument, Helsingborg, Sweden). The sections were mounted on 3′-aminopropyltriethoxysilane (APES; Merck KGaA, Darmstadt, Germany) coated glass slides and stained as described by Warwas et al. [45] using hematoxylin (Histolab Products AB, Askim, Sweden), eosin (Histolab Products AB, Askim, Sweden), and alcian blue 8 GX (pH 2.5, Merck, KGaA, Darmstadt, Germany). Images of the sections were produced using a 2.3 MP camera (PowerPack ace 2.3 MP, Basler AG, Ahrensburg, Germany) mounted to an Eclipse E1000 (Nikon, Tokyo, Japan) microscope at tenfold magnification. Two pictures of each cross-section were taken, and the resulting 12 images per fish and intestinal region were analyzed for villi length, lamina propria width, submucosa width, and goblet cell count based on the indicators described by Baeverfjord and Krogdahl [46] and modified by Warwas et al. [45] using the ImageJ software (Wayne Rasband, NIH, USA). The supranuclear vacuolization was scored according to Knudsen et al. [16]. Six fish were analyzed for each dietary treatment.

### 2.9. Plasma Cortisol, Ghrelin, and Glucose

Plasma cortisol was analyzed according to [47] using a cortisol antibody validated by Sundh et al. [48] (Code: S020; Lot: 1014-180182, Guildford Ltd., Guildford, UK). Hydrocortisone [1,2,6,7-^3^H (N)], NEN Life Sciences Products, Boston, USA) was used as a tracer. Concentrations were determined using a standard curve of cold cortisol (0.5–512 ng mL^−1^, Sigma, St. Louis, MO, USA). Radioactivity was measured in a Wallac 1409 liquid scintillation counter (LKB Instruments, Turku, Finland). Plasma ghrelin levels were analyzed using a radioimmunoassay according to Hosoda et al. [49], with modifications by Jönsson et al. [50], using an antibody kindly provided by Dr. Hiroshi Hosoda (Osaka, Japan). Human ^125^I-ghrelin was used (Merck KGaA, Darmstadt, Germany) as a tracer. The glucose concentration was determined using an enzyme assay kit (Glucose GHK, Sigma, St. Louis, MO, USA). A cryoscopy osmometer was used to analyze plasma osmolarity (Advanced Model 3320 Micro-Osmometer, Advanced Instruments Inc., Norwood, MA, USA).

### 2.10. Gene Expression

Gene expression analysis was carried out as described by Warwas et al. [38, 45]. Between 20 and 30 mg of tissue was homogenized mechanically using one inert steel bead using the TissueLyser II (Qiagen NV, Qiagen NV, Hilden, Germany). The homogenate was buffered in 600 μL lysis buffer (RLT plus buffer), and hypothalamic RNA was extracted using the RNeasy Plus Mini Kit (Qiagen NV, Hilden, Germany). cDNA was synthesized using the iScript cDNA Synthesis Kit (Bio-Rad Laboratories Inc., Richmond, CA, USA). Samples with RNA concentrations < 20 ng μL^−1^ were excluded from the analysis. Real-time quantitative PCR (RT-qPCR) was used to quantify mRNA concentrations and determine relative gene expression. RT-qPCR reactions were carried out in duplicates using a primer concentration of 0.5 µM and the SsoAdvanced Universal SYBR Green Supermix (Bio-rad Laboratories Inc., Richmond, CA, USA). Primer pairs of target and reference genes can be found in Table 2. The RT-pPCR reaction was carried out over 40 cycles with 95°C denaturation and 60°C annealing and extension (CFX96 Connect Real-time PCR Detection system Bio-Rad Laboratories Inc., USA). RNA quality, as well as primer efficiency, product purity, and product size, were confirmed as described in Warwas et al. [38]. Relative gene expression of the target genes was calculated using the 2−ΔCT' method ([54], Equation (4)).

### 2.11. Calculations

The MCHC was calculated by dividing Hb with Hct (Equation (1)). Using liver, viscera, and whole-body weight, HSI (Equation (2)) and VSI (Equation (3)) were calculated. Target gene expression was calculated using the 2−ΔCT' method (Equation (4)). Weight gain (WG) was calculated by subtracting IBW from final body weight (FBW). The specific growth rate (SGR) was calculated using IBW, FBW, and the experimental period in days (*d*, Equation (5)). Using fork length (L) and FBW, the condition factor was calculated (Equation (6)). By dividing the dry weight of the consumed feed (*F*) with the WG, the feed conversion ratio (FCR) was calculated (Equation (7)). Survival was calculated using the initial number of individuals (*ni*) and the number of individuals at the final sampling day (*nf*, Equation (8)). The apparent permeability of mannitol (Papp, Equation (9)) was calculated using the appearance rate of ^14^C-mannitol (*dQ/dt*) on the serosal side and the initial ^14^C-mannitol concentration. The uptake rate of ^3^H-L-lysine (Equation (9)) was calculated using the appearance rate of ^3^H-L-lysine and the ratio of labeled and unlabeled L-lysine (Equation (10)).

(1) $$\begin{array}{c} \text{MCHC}=\text{ Hb}/\text{Hct }\times 10 \end{array}$$

(2) $$\begin{array}{c} \text{HIS }\left(\text{\% }\right)=\text{Liver weight}/\text{FBW }\times 100 \end{array}$$

(3) $$\begin{array}{c} \text{VSI }\left(\text{\% }\right)=\text{Viscera weight}/\text{FBW }\times 100 \end{array}$$

(4) $$\begin{array}{c} \text{Rel}.\text{ expression }={2_{T}}^{-\left(C_{T}\left(\text{reference}\right)-C_{T}\left(\text{target}\right)\right)} \end{array}$$

(5) $$\begin{array}{c} \text{SGR }\left(\text{\% BW }d^{-1}\right)=\left(\ln\text{ FBW }-\ln\text{ IBW}\right)/d\times 100 \end{array}$$

(6) $$\begin{array}{c} \text{Condition factor }=\left(\text{FBW }/L^{3}\right)\times 100 \end{array}$$

(7) $$\begin{array}{c} \text{FCR}=\text{ FI}/\text{ WG} \end{array}$$

(8) $$\begin{array}{c} \text{Survival }\left(\%\right)=nf/ni\times 100 \end{array}$$

(9) $$\begin{array}{c} \text{Papp}\left(\text{cm }s^{-1}\right)=dQ/dT\times 1/A\times C0 \end{array}$$

(10) $$\begin{array}{c} \text{Lysine }\left(\text{mol }\times \min^{-1}\times {\text{cm}}^{-2}\right)=\left(dQ/dT\right)\times \left(1/A\right) \end{array}$$

### 2.12. Statistical Analysis

For the data analyses, SPSS 29 (SPSS Inc., Chicago, IL, USA) was used. Data normality was tested for using a Shapiro–Wilk test as well as a visual inspection of the residual Q–Q plots. Homogeneity was analyzed using Levene's test. Statistical differences between groups were determined using a nested analysis of variance (ANOVA) with “tank” and “diet” as fixed factors, where the tank was nested within the treatment. Thus, the tank was treated as an experimental unit (*n* = 3). Results of the histological evaluation of the intestine were analyzed using a one-way ANOVA with fish as an experimental unit (*n* = 6). When significant results were obtained by the ANOVA, Tukey's post-hoc test, adjusted for multiple comparisons, was used for pairwise comparisons between groups. In cases where the ANOVA assumptions were violated, the data set was transformed using a log_10_ transformation to meet the requirements. If assumptions were still not met, a nonparametric Kruskal–Wallis followed by Dunn's post hoc test was performed. Significant differences between groups were assumed at *p* < 0.05. All values are presented as means ± SD.

## 3. Results

### 3.1. Raw Materials and Diets

The proximate and amino acid compositions of the insect meals, as well as the fish meal, can be found in Table 3. The SWFL contained 60% protein, including 3.5% lysine and 1.2% methionine, which was higher than what was found for the defatted BSFL (57%, 1.6%, and 0.5%, respectively) and slightly lower than that of commercial fish meal (67%, 5.3%, 2%, Table 3). The lipid content of the SWFL was 17%, about 1% lower than what was found for the partially defatted BSFL meal (18%) and higher than that of fish meal (*ca*. 11%). Additionally, the fatty acid profile of the SWFL contained 3.4% EPA and 0.2% DHA, resulting in an omega *n*-3 to omega *n*-6 ratio of 0.9 (Table 4). The chemical composition of the three experimental diets can be found in the supplement (Table S1 and Table S2).

### 3.2. Growth and Feed Utilization

**Figure 1:**
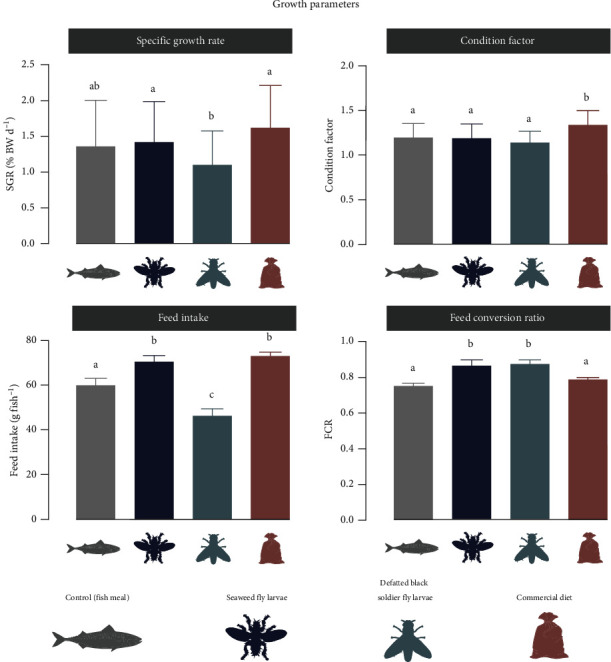
Specific growth rate (SGR), condition factor, feed intake, and feed conversion ratio (FCR) of rainbow trout fed one of four experimental feeds, control, seaweed fly larvae, partially defatted black soldier fly larvae, and commercial reference diet. Bars indicate mean ± SD. The letters above bars indicate significant differences between the groups, *p*-values < 0.05.

Numerically, the highest SGR was observed for the fish fed the reference diet (1.62 ± 0.59% d^−1^), followed by fish fed the SWFL (1.42 ± 0.56% d^−1^) and control diets (1.36 ± 0.64% d^−1^, Figure 1). Both the reference and the SWFL diet-fed fish displayed significantly higher growth rates compared to fish fed the BSFL diet (1.10 ± 0.48% d^−1^). FI was also higher in fish fed the SWFL (70.3 ± 2.9 g) and reference diets (72.8 ± 1.9 g) compared to the control (59.7 ± 3.3 g) and BSFL (46.1 ± 3.3 g) diets. FCR was lower for control (0.75 ± 0.02) and reference (0.79 ± 0.01) diet-fed groups compared to the SWFL (0.86 ± 0.04) and BSFL (0.87 ± 0.03) diet-fed fish. The condition factor was highest for fish fed the Ref diet (1.33 ± 0.16), while no differences were found between the three experimental diets (Table 5). HSI was lowest for the reference diet (1.29 ± 0.23), while no difference was found for the three experimental diets. None of the dietary treatments had any effect on VSI or survival.

### 3.3. Fish Whole Body Composition

No differences in macronutrient, energy, or ash content were observed for the whole-body composition of fish from the different treatments (Table 6). Additionally, there were no differences in the amino acid content between the control, BSFL, and SWFL diets, while fish fed the Ref diet displayed higher arginine levels compared to fish fed the SWFL diet. While total lipid levels of the whole body were unaffected, the fatty acids composition varied slightly. Fish fed the SWFL diet displayed higher levels of palmitoleic acid (C16 : 1 *n*-7, 4.9% ± 0.3% of total fatty acids) compared to the other diets (C: 3.4% ± 0.3%, BSF: 3.2% ± 0.4% and Ref: 3.0% ± 0.4%). Additionally, fish fed the SWFL diet displayed higher levels of arachidonic acid (C 20 : 4 *n*-6, 0.4% ± 0.1%) compared to fish given the Ref diet (0.2% ± 0.1%, Table 6). While not significantly different, fish fed the SWFL diet displayed numerically higher levels of both DHA and EPA compared to the fish fed BSFL as well as the Ref diet (Table 6).

### 3.4. Appetite Regulation

The mRNA concentration of genes coding for neuropeptides connected to central appetite regulation was overall low and no effects of any of the diets were observed (Table 7). Plasma ghrelin levels were numerically highest in the BSFL-fed fish (34.16 ± 16.6 pmol L^−1^), but no statistically significant differences between the treatment groups were observed.

### 3.5. Intestinal Health

#### 3.5.1. Intestinal Barrier and Transport Functions

**Figure 2:**
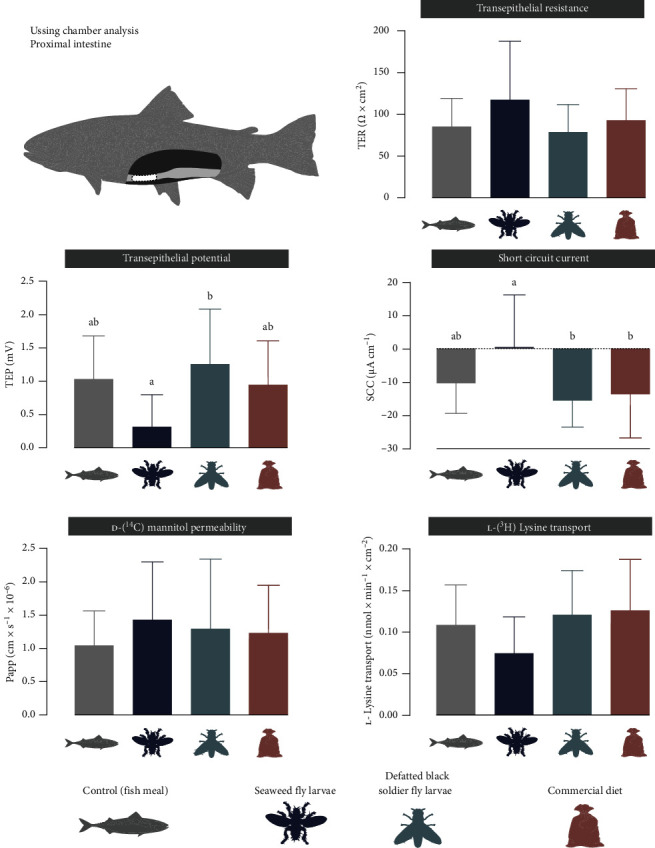
Ussing chamber measurements of transepithelial resistance (TER), transepithelial potential (TEP), short circuit current (SCC), permeability for ^14^C-mannitol (Papp), and transport rate of ^2^H-lysine in the proximal intestine of fish fed one of four experimental feeds, control, seaweed fly larvae, partially defatted black soldier fly larvae and commercial reference diet. Bars indicate mean ± SD. The letters above bars indicate significant differences between the groups, *p*-values < 0.05.

**Figure 3:**
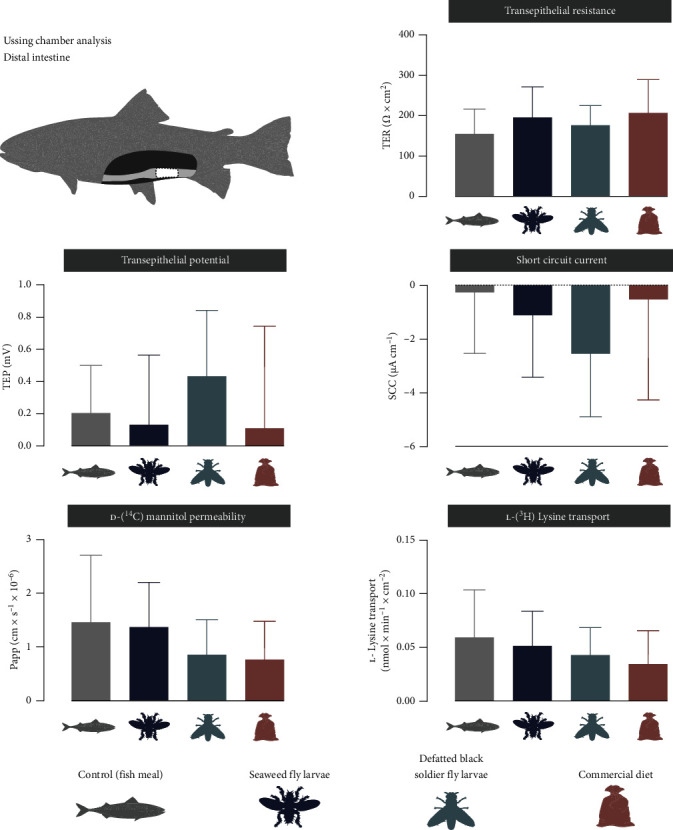
Ussing chamber measurements of transepithelial resistance (TER), transepithelial potential (TEP), short circuit current (SCC), permeability for ^14^C-mannitol (Papp), and transport rate of ^2^H-lysine in the distal intestine of fish fed one of four experimental feeds, control, seaweed fly larvae, partially defatted black soldier fly larvae and commercial reference diet. Bars indicate mean ± SD. The letters above bars indicate significant differences between the groups, *p*-values < 0.05.

In the proximal intestine, fish fed the SWFL diet displayed a lower TEP (0.32 ± 0.48 mV) and a lower (in absolute value) SCC (0.52 ± 15.6 µA cm^−2^) compared to fish fed the BSFL diet (1.26 ± 0.8 mV and −15.6 ± 7.9 µA cm^−2^, respectively, Figure 2). Additionally, the absolute value of SCC for fish fed the SWFL diet was lower than that of the Ref treatment (−13.7 ± 13.2 µA cm^−2^). No differences in TER, Papp, or lysine transport were observed in the proximal intestine. For the distal intestine, no significant differences were found between the diets (Figure 3).

#### 3.5.2. Histological Parameter

No significant differences in the intestinal morphology in either proximal or distal intestine were observed between fish fed the SWFL, BSFL, and control diets (Table 8). The fish fed the Ref diet displayed significantly wider lamina propria width in the proximal intestine (17.3 ± 2.2 µm) compared to fish fed SWFL (13.3 ± 1.7 µm), BSFL (13.6 ± 2.7 µm), and control diet (13.3 ± 1.7 µm).

### 3.6. Hematological Parameters

Diet had a significant effect on Hct and Hb plasma levels (Table 9). The fish-fed SWFL and Ref diets displayed the highest levels of Hb (7.18 ± 1.0 and 8.02 ± 1.1 g dL^−1^, respectively). Fish fed the control diet displayed significantly lower Hb values than both diets (6.19 ± 1.6 g dL^−1^). Fish-fed BSFL diet displayed lower Hb values (6.50 ± 1.29 g dL^−1^) compared to the Ref diet. Hct levels were higher for fish fed the SWFL (35.8% ± 4.3%) and Ref diets (38.7% ± 5.6%) compared to the control (32.0% ± 7.9%) and BSFL (32.9% ± 8.3%) diets. No difference between the treatment groups was found for MCHC, plasma glucose, plasma cortisol, or plasma ghrelin.

## 4. Discussion

### 4.1. Rearing Conditions and Substrates

The first objective of the present study included the cultivation of SWFL using two algal species (*F. vesiculosus* and *S. latissima*). SWFL effectively converted the biomass derived from the two marine algae into nutrient-dense biomass. This is consistent with a previous study cultivating *C. frigida* using two different but closely related brown algae species, *Laminaria digitata* and *Fucus serratus* [37]. The substrate requirements and the life cycle of SWFL differ markedly from those of the BSFL [55]. BSFL can be reared efficiently on a wide variety of organic side streams, including household food waste, manure, chicken feed, and bread [21, 55–57]. However, marine substrates such as algae appear to be less optimal substrates for BSFL. The cultivation of BSFL on *Ascophyllum nodosum* (brown algae) and *Schizochytrium sp*. (microalgae) has been shown to reduce growth rates and survival of the larvae [32, 35]. SWFL, on the other hand, naturally grows on marine macroalgae and may, therefore, be more efficient at incorporating marine nutrients such as LC-PUFAs from marine substrates compared to BSFL.

The production cycles of SWFL and BSFL differ markedly, which has implications for their commercial application. The complete life cycle of SWFL in this experiment was between 10 and 14 days, including 1 week of larval growth phase and 1 week of pupae, adult stage, and hatching. Larvae cultures can, therefore, be harvested weekly. *H. illucens*, on the other hand, take roughly 30 days to complete their life cycle [55]. The faster life cycle of SWFL suggests a higher efficiency in terms of time, space, and, therefore, logistical benefits compared to the BSFL, as two batches of SWFL can be produced for every batch of BSFL. Additionally, the short life cycle will reduce the lag in production of the first batch when rearing SWFL, which may enable efficient adjustments to changes in product demand. On the other hand, BSFL are considerably larger (*ca*. 70 mg, [55]) compared to the SWFL (average harvest size of 45 mg in the present study), largely due to the high accumulation of lipid in BSFL.

### 4.2. Nutrient Composition

Fish oil and fish meal have traditionally been the main sources of EPA and DHA for the salmonid aquaculture industry [2]. Due to the limited access to marine raw materials as well as ethical considerations regarding capture fisheries and the utilization of human food grade fish for fish feed production, these essential omega *n*-3 fatty acids, but also essential amino acids, such as lysine and methionine, have become bottlenecks in the feed sector [58, 59]. SWFL contained 3.5% lysine and 1.2% methionine, which is slightly lower compared to fish meal but higher compared to the partially defatted BSFL used in this study, as well as many plant-based ingredients [60]. On an amino acid basis (% of amino acids), the SWFL meal was comparable to a commercial fish meal, with *ca*. 8% of amino acids being lysine and *ca*. 3% being methionine. The SWFL meals CP of 60% was comparable to partially defatted BSFL meal and slightly lower than that of fish meal (*ca*. 70%; [8, 57]). Insect meal contains considerable levels of non-protein nitrogen as part of chitin, likely resulting in an overestimation of the total protein in both SWFL and BSFL meals. Both insect meals displayed a similar total amino acid content, 43%–44%, which was slightly lower than that of fish meal. As tryptophan and tyrosine were not part of the chemical analysis, the actual protein consent of SWFL may be 45%–60%. Notably, both fish meal and defatted BSFL meal represent a separated protein fraction of whole fish and larvae, respectively (the other major fraction being fish and insect oils). In extruded diets, the lipid content of the final mash may not exceed 5%–10%, depending on the extruder to ensure the sheer forces and expansion needed to form stable pellets [61]. Thus, the high protein content and comparatively low lipid content of SWFL have positive implications for processing and feed production, as, unlike most BSFL meals, an additional separation of lipid and protein fractions is expendable. Such additional processing steps (e.g., pressing, heating, and purifying) to create protein meals result in additional costs, energy inputs, and equipment. The SWFL also contained considerable amounts of LC-PUFAs, mainly in the form of EPA (3.4% of fatty acids). While this is a bit lower than in fish meal, terrestrial insects, including BSFL, naturally do not contain either DHA or EPA [62]. In contrast, insects associated with the marine environment, such as SWFL, have been shown to naturally contain omega-3 fatty acids, especially EPA [36, 37]. In line with these findings, the SWFL meal evaluated in the present study contained 17% lipids of which 13% were PUFAs, and 3.4% of these were EPA. Fish meal contains *ca*. 11% lipids, of which roughly 40% are PUFAs [8]. With regard to carnivorous fish, SWFL meal may, therefore, be nutritionally more complete than many commercial BSFL meals where insects are reared on terrestrial substrates. Furthermore, the ash content of the SWFL (13%) was comparable to that of fish meal (10%–15%) and double that of the defatted-BSFL meal used in this study. This was likely due to the higher salt and mineral content in the substrate (marine algal biomass) used for the SWFL cultivation as compared to most commercial BSFL substrates [57, 63]. Ash resembles a diverse mix of minerals, many of which are essential micronutrients such as phosphate, selenium, and calcium. Too high levels of ash, however, may have adverse effects, not least because the mineral fraction does not contribute to energy or macronutrients [64]. The similar ash content of SWFL meal and fish meal is therefore encouraging.

The nutrient composition of farmed insects is highly influenced by, and can thus be modulated through, the cultivation substrate [20, 23]. For this reason, marine side streams, including fish, mussels, and algae, have been added to cultivation substrates in order to increase the nutritional value and, specifically, the content of DHA and EPA of BSFL [19, 21, 30–33, 35]. LC-PUFAs are associated with an array of benefits for salmonid health and welfare, including intestinal barrier and transporting function [58, 65]. Additionally, the inclusion of 30% BSFL, cultured on terrestrial side streams alone, has been shown to decrease the content of omega-3 fatty acids in rainbow trout filets, thereby reducing the human health benefits [66, 67]. Both the lower filet LC-PUFA content as well as the risk for compromised animal health and welfare have been the main drivers in the quest to improve the fatty acid profile of BSFL. However, despite the potential to positively affect the fatty acid profile, high marine substrate inclusion also reduced growth, survival, and protein content in BSFL [21, 32, 35]. These observations suggest a tradeoff between production efficiency and optimal fatty acid profile and clearly indicating that marine substrates such as algae are suboptimal for BSFL rearing. The SWFL in the present study, on the other hand, grown on brown algae substrates, displays high growth rates and high levels of proteins while maintaining high omega-3 levels, which is in line with previous studies [37]. Therefore, compared to BSFL, SWFL appears to be a more suitable vector for the conversion of marine side streams, particularly macroalgae, into a high-quality feed ingredient for marine fish.

In summary, the nutritional composition of the SWFL meal may align more with the nutrient requirements of carnivorous fish compared to that of terrestrial insects as well as that of many plant-based protein sources [60, 68]. This alignment may permit high inclusion levels in diets for salmonid fish without compromising the nutritional quality of both the feed and the final product that is the fillet. SWFL show great potential to efficiently utilize and transform marine side streams into protein-rich biomass and thus may contribute to a more resource-efficient feed and food production.

### 4.3. Effects of Dietary SWFL Inclusion on Growth Physiology

The second objective of the present study was to evaluate SWFL as a protein source in diets for rainbow trout. Substituting 40% of the fish meal with SWFL meal had no negative effect on either WG, growth rate (SGR), or condition factor. Fish fed the SWFL diet grew as well as fish fed both the control diet and the commercial reference diet and displayed, after the reference diet, the highest FI and SGR. Fish fed the BSFL diet, on the other hand, displayed a lower FI and SGR compared to the SWFL and reference diets. Despite being a high-quality protein source and outperforming various plant-based ingredients regarding nutrient profile and maximum inclusion level in diets for salmonid fish, replacing large fractions of fish meal (>30% gross inclusion) with BSFL meal has been shown to reduce FI and growth in both rainbow trout and Atlantic salmon [26–28, 69]. Additionally, insect protein has been shown to increase FCR when substituting fish meal, suggesting a comparatively lower bioavailability [27, 28]. In line with these observations, the FCR of both SWFL and BSFL was elevated compared to the control and reference diets in the present study. The increase in FCR may partly be due to the insects' chitin content, which has been suggested to have antinutritional properties, resulting in decreased nutrient digestibility and growth in rainbow trout [27, 70]. While not explicitly measured in this study, chitin, due to its structural similarities with cellulose, may be estimated as the major part of the NDF fraction in insect meals [71]. The lower NDF levels in the SWFL meal compared to the defatted BSFL meal used in the present study, therefore, suggest a lower chitin content in SWFL. One reason for the potentially lower chitin content of the SWFL may be the manual removal of pupae and substrate/from larvae postharvesting. The chitin content of BSFL and SWFL meals may, therefore, be more similar in similar production systems (manual vs. automated). On the other hand, chitin, which contains nitrogen, may also roughly be estimated through the discrepancy of the CP content (based on total nitrogen) and the sum of the amino acids. Since both CP and the sum of amino acids were similar between SWFL and defatted BSFL meals, the chitin content of the two meals may be more similar than suggested by the NDF fraction. However, it is still likely that a defatted BSFL meal will maintain a proportionally higher chitin content due to the additional lipid removal, which may result in not only a proportionally higher protein but also chitin content.

FCR was similar for both insect diets, whereas the FI was significantly higher for the SWFL diet compared to the BSFL diet. Even though no difference in the expression of appetite-regulating neuropeptides was observed between the four diets, the increase in FI suggests a higher palatability of the SWFL. As a result, the observed higher FI compensated for the possibly lower bioavailability of insect meal compared to fish meal and resulted in growth rates comparable to both control and commercial reference feeds. Various substances have been identified to stimulate FI in rainbow trout, including specific amino and fatty acids [64]. For instance, rainbow trout have been shown to preferentially choose diets with elevated levels of DHA and EPA [72]. As commercial BSFL meals contain low or no LC-PUFAs, the presence of considerable levels of EPA present in the SWFL may contribute to the higher palatability and, thus, the increased FI and growth. In addition, alanine, one of the FI-stimulating amino acids, was higher in the SWFL meal compared to the defatted BSFL meal [73–75]. Still, the experimental diets were formulated to be isonitrogenous, and as a result, the amino acid profiles of the three diets displayed very small variations, which are unlikely to be the main driver for the large differences in FI.

Despite not being significant, the whole-body composition analysis revealed that fish fed the SWFL diet contained LC-PUFAs in levels that were in the same range as those of fish that received the control diet, whereas fish fed BSFL and the reference diets had lower levels. Additionally, the SWFL diet resulted in a higher level of palmitoleic acid (C16:1n7) compared to the other three diets, which is in line with previous studies demonstrating that insect meal can increase the short-chain fatty acid contents in fillets of rainbow trout [66, 67]. Still, the higher LC-PUFA content in fish fed the SWFL diet is highly promising.

In summary, SWFL meal appear to be highly palatable, which may be influenced by both a favorable fatty acid profile due to the presence of LC-PUFAs and a balanced amino acid profile. The resulting high FI of fish fed the SWFL diet yielded high growth rates, which were comparable to the fish meal control and commercial reference diet. Substituting fish meal with either SWFL or BSFL resulted in an increase in the FCR, likely due to a lower bioavailability.

### 4.4. Health and Welfare

An assessment of potential effects on general animal health and welfare of novel feed ingredients is essential [8]. While being a highly promising ingredient, studies have identified negative effects of high BSFL inclusion (>25%) on the intestinal health of several fish species, including rainbow trout [69, 76, 77]. An assessment of intestinal health in response to dietary SWFL inclusion was, therefore, a central part of the present study. Fish fed the SWFL diet displayed lower TEP and SCC (in absolute values) compared to fish fed the BSFL diet. As TEP and SCC are indicators for active transport in the intestinal epithelium, reduced levels can be associated to lower nutrient uptake and negative downstream effects on health and growth. However, fish fed the SWFL displayed higher SGR and FI compared to the BSFL, and the analysis of the histopathological parameters showed no indication of an intestinal inflammation. The changes in SCC and TEP, therefore, may reflect an equally efficient, less electrogenic uptake of amino acids in the SWFL-fed fish [78]. This is supported by the measured intestinal lysine uptake, which did not differ between the dietary treatments. Thus, amino acids were likely taken up at similar rates, potentially involving less energy for electrogenic transport, such as sodium-coupled amino acid uptake. Additionally, the intestinal electrophysiological parameters of fish fed either SWFL or BSFL were not significantly different from those of fish fed the fish meal-based control diet, further supporting the notion of good intestinal health.

The Hb and Hct values displayed by fish fed the SWFL diet were more comparable to the values of fish fed the commercial reference diet rather than either the control or the BSFL diets. The slightly higher Hb levels of SWFL-fed fish compared to the control may reflect an increase in red blood cell production in response to lower oxygen levels. However, oxygen levels were monitored throughout the experiment, and hypoxic conditions were avoided. Additionally, Hb levels of fish fed the SWFL were similar to those of fish fed the commercial reference diet and within the normal range for healthy, undisturbed rainbow trout [79–82]. The fluctuations in Hb values between the diets may thus not reflect changes in animal health and welfare but rather the nutritional and mineral makeup of the diets. Potential relationships between the SWFL diet and, for example, increased oxygen-carrying capacity or fluid balance should be further investigated prior to drawing concrete conclusions.

In summary, the tested diets resulted in good health and welfare. Interestingly, the two insect meals differed in their effect on the intestinal electrophysiology, highlighting the heterogenous properties of different insect products.

## 5. Conclusion

This study demonstrates that “marine” insects such as the seaweed fly larvae are a promising vector to retain marine nutrients, for example, from side streams of the seaweed and fishery industry, in the food production chain. This is especially valuable considering the aquaculture industry's need for sustainable alternative marine ingredients due to the finite access to fish meal and oil. The SWFL displayed 60% CP and contained high levels of essential amino acids, including lysine and methionine, as well as significant levels of LC-PUFAs. Therefore, marine insects may be nutritionally advantageous compared to terrestrial insects. Furthermore, SWFL had an FI stimulating effect suggesting the possibility to utilize marine insects as a strategic functional feed ingredient. Future studies evaluating the potential upscaling of seaweed fly production systems, the potential functional properties, and digestibility are encouraged to aid this development.

## Figures and Tables

**Table 1:** Feed formulation, proximate composition, dry matter (DM), and gross energy content on DM basis in g/100 g of the four experimental diets, control (C), seaweed fly larvae (SWFL), black soldier fly larvae (BSFL), and commercial reference diet (Ref).

Ingredient	C	SWFL	BSFL	Ref^a^
Fish meal^b^	25.00	15.00	15.00	—
Soy protein concentrate^c^	16.00	18.00	18.00	—
Wheat gluten^d^	13.40	14.00	14.00	—
Wheat meal	14.00	14.00	14.00	—
Fish oil^e^	10.00	10.00	10.00	—
Rapeseed oil	9.00	8.44	8.44	—
Vitamin and mineral premix	1.80	1.80	1.80	—
Faba bean meal	8.90	4.00	4.14	—
Seaweed fly larvae meal	0.00	12.76	0.00	—
Defatted black soldier fly larvae meal^f^	0.00	0.00	12.57	—
Proximate composition				
Dry matter (%)	95.43	91.29	93.63	
Crude protein^g^	46.0	45.79	47.10	42–45
Crude lipid^h^	22.21	21.60	25.08	24–27
Ash	6.61	5.85	6.61	4.5–6.5
Neutral detergent fiber	2.97	3.55	6.69	0.8–2.3^i^
Gross energy (MJ/kg DM)^j^	23.2	23.4	24.0	23.5–25.5

^a^Biomar Efico Enviro 921 Advance, Aarhus, Denmark, values refer to pellets as is according to MioMar.

^b^Low-temperature fish meal, North Atlantic origin.

^c^Hamlet, Horsens, Denmark.

^d^Lantmännen Reppe, Lidköping, Sweden.

^e^North Atlantic origin.

^f^Protix Biosystems BV, Dongen, The Netherlands.

^g^According to Kjeldahl (N x 6.25).

^h^According to Schmid–Bondzynski–Ratslaff.

^i^Crude cellulose, according to BioMar.

^j^Using bomb calorimetry.

**Table 2:** Nucleotide sequence of primers used to evaluate mRNA concentration of target genes appetite regulation in the hypothalamus of juvenile rainbow trout.

Gene	Direction	Sequence	Accession number	References
CART	F	GTCCATCGTTCTTAGTGCTGAA	AB455538	[51]
	R	CAG TTGCTTTTCGTT GGTCAA		
CRF	F	ACAACGACTCAACTGAAGATCTCG	NM001124286	[51]
	R	AGGAAATTGAGCTTCATGTCAGG		
MC4R	F	TTCTCACACTGGGGATAGTCA	AY534915.1	[51]
	R	CACAGCCAAAGAACAGATGAAT		
NPY	F	AGAATTGCTGCTGAAGGAGAG	AF203902	[51]
	R	GGGACAGACACTATTACCACAA		
POMCA1	F	CTCGCTGTCAAGACCTCAACTCT	TC86162	[52]
	R	CAATAACCACGCAGGACACA		
Ubiquitin	F	AGATAAATCGGAGAGTTGCTGTG	X99303	[53]
	R	CCTGCTCCACCTTGTGTTGT		
*β*-Actin	F	ATGGAAGATGAAATCGCC	AF157514	—
	R	TGCCAGATCTTCTCCATG		

Abbreviations: CART, cocaine- and amphetamine-regulated; CRF, corticotropin-releasing factor; F, forward; MC4R, melanocortin 4 receptor; NPY, neuropeptide Y; POMCA1, proopiomelanocortin A1; R, reverse.

**Table 3:** Proximate and amino acid composition on a dry matter basis (g/100 g) of fish meal, *C. frigida* larvae, and *H. illucens* larvae.

Composition	Fish meal^a^	*C. frigida*	*H. illucens* ^b^
Dry matter (%)	91.8	92.7	97.6
Crude protein^c^	67	60.0	56.5
Sum amino acids	62	44.1	43.0
Crude lipid^d^	10.9	16.7	17.8
Gross energy^e^	20.1	22.8	24.4
Ash	13.9	13.2	7.4
Neutral detergent fiber	—	7.55	35.3
*Indispensable amino acids*			
Arginine	3.9	2.5	1.4
Histidine	2.5	1.5	0.8
Isoleucine	3	2.1	1.2
Leucine	5	3.1	2.0
Lysine	5.3	3.5	1.6
Methionine	2	1.2	0.5
Phenylalanine	2.7	2.7	1.3
Threonine	2.5	2.2	1.2
Valine	3.7	2.7	1.7
*Dispensable amino acids*			
Alanine	4.1	4.2	2.0
Aspartic acid	5.8	4.3	2.8
Cysteine	0.9	0.5	0.3
Glutamic acid	8.3	6.7	3.1
Glycine	4.3	2.4	1.6
Proline	2.7	2.5	1.6
Serine	2.2	1.9	1.2

^a^Anchoveta meal, TASA, Peru, data are adopted from Glencross, 2020.

^b^Protix Biosystems BV, Dongen, The Netherlands.

^c^According to Kjeldahl (*N* x 6.25).

^d^According to Schmid–Bondzynski–Ratslaff.

^e^Using bomb calorimetry.

**Table 4:** Fatty acid composition in % of total fatty acids of seaweed fly larvae, *C. frigida*.

Composition	*C. frigida*
C 12:0	0.5
C 14:0	7.4
C 14:1 *n*-5	1.3
C 15:0	0.6
C 16:0	17.8
C 16:1 *n*-7	31.1
C 17:0	0.4
C 17:1 *n*-7	0.9
C 18:0	1.7
C 18:1	17.3
C 18:2 *n*-6	3.3
C 18:3 *n*-3	1.6
C 18:3 *n*-6	0.5
C 18:4 *n*-3	1.6
C 20:0	0.2
C 20:4 *n*-6	2.4
C 20:4 *n*-3	0.1
C 20:5 *n*-3 (EPA)	3.4
C 22:0	0.1
C 22:6 *n*-3 (DHA)	0.2
Total saturated fatty acids	28.9
Total single unsaturated fatty acids	50.7
Total polyunsaturated fatty acids	13.3
Total omega *n*-6 fatty acids	6.3
Total omega *n*-3 fatty acids	7.0
Ratio omega *n*-6/omega *n*-3 fatty acids	0.90

**Table 5:** Growth parameters and body indices of rainbow trout fed one of the experimental diets; control (C), seaweed fly larvae (SWFL), black soldier fly larvae (BSFL), and reference diet (Ref).

Parameter	Diet				*p*-Value	
	C	SWFL	BSFL	Ref	Diet	Tank
Initial weight (g) (*n* = 18)	40.74 ± 8.6	40.93 ± 8.03	40.65 ± 8.28	40.65 ± 8.53	0.998	0.941
Final weight (g) (*n* = 18)	116.9 ± 43.6^a^	119.6 ± 46.0^a^	93.5 ± 43.6^b^	137.7 ± 50.2^a^	<0.001	0.992
Condition factor (*n* = 18)	1.19 ± 0.16^a^	1.19 ± 0.16^a^	1.14 ± 0.13^a^	1.33 ± 0.16^b^	<0.001	0.408
Total feed offered (g fish^−1^, *n* = 3)	67.26 ± 3.7^a^	76.72 ± 3.7^b^	56.36 ± 2.8^c^	76.69 ± 1.3^b^	<0.001	—
Feed waste (%, *n* = 3)	11.24 ± 1.66^a^	8.35 ± 1.37^a^	19.71 ± 5.57^b^	6.89 ± 1.46^a^	0.004	—
HSI (*n* = 4)	1.57 ± 0.20^a^	1.73 ± 0.46^a^	1.79 ± 0.28^a^	1.29 ± 0.23^b^	0.005	0.383
VSI (*n* = 4)	10.53 ± 2.25	12.82 ± 4.30	11.08 ± 1.65	11.39 ± 1.54	0.178	0.200
Survival (%) (*n* = 3)	100.0 ± 0.0	100.0 ± 0.0	98.13 ± 3.23	98.13 ± 3.23	0.596	—

*Note*: The data are shown as mean ± SD. Values within rows with different superscripts are significantly different (*p* < 0.05).

**Table 6:** Whole body composition (macronutrients in g/100 g DM, energy in MJ/kg, and fatty acids in % of total fatty acids) of rainbow trout fed one of the experimental diets; control (C), seaweed fly larvae (SWFL), black soldier fly larvae (BSFL), and the commercial reference diet (Ref).

Parameter	Diet				*p* -Value
	C	SWFL	BSFL	Ref	
Crude protein	54.71 ± 1.16	52.16 ± 2.30	54.61 ± 2.30	56.04 ± 2.91	0.195
Crude lipid	36.27 ± 2.76	37.48 ± 2.57	36.27 ± 2.77	34.41 ± 0.50	0.495
Carbohydrates	1.22 ± 1.62	2.63 ± 1.92	1.39 ± 1.00	1.95 ± 2.13	0.751
Gross energy	22.93 ± 5.79	23.19 ± 5.59	22.94 ± 6.44	22.59 ± 0.79	0.591
Ash	7.65 ± 0.19	7.58 ± 0.29	7.59 ± 0.78	7.46 ± 0.42	0.966
C 16:1 *n*-7	3.40 ± 0.26^a^	4.90 ± 0.26^b^	3.23 ± 0.38^a^	3.00 ± 0.44^a^	0.001
C 20:4 *n*-6	0.27 ± 0.06^ab^	0.43 ± 0.12^a^	0.27 ± 0.06^ab^	0.23 ± 0.06^b^	0.047
C 20:5 *n*-3 (EPA)	1.53 ± 0.64	1.63 ± 0.46	1.23 ± 0.15	1.20 ± 0.17	0.517
C 22:6 *n*-3 (DHA)	5.53 ± 2.56	5.73 ± 1.68	4.33 ± 0.75	4.03 ± 0.68	0.511
Omega *n*-6 fatty acids	13.90 ± 1.14	14.27 ± 0.49	14.97 ± 0.40	15.40 ± 0.44	0.101
Omega *n*-3 fatty acids	12.43 ± 4.65	12.97 ± 2.93	10.87 ± 1.05	10.90 ± 0.40	0.732
Omega *n*-6/*n*-3 ratio	1.24 ± 0.47	1.14 ± 0.25	1.39 ± 0.15	1.41 ± 0.08	0.602

*Note*: The data are shown as mean ± SD. Values within rows with different superscripts are significantly different (*p* < 0.05). Crude proteins was analyzed according to Kjeldahl (*N* x 6.25), Crude lipid was analyzed according to Schmid–Bondzynski–Ratslaff, Gross energy was analyzed using bomb calorimetry.

**Table 7:** Gene expression (x 10^−3^) relative to ubiquitin and *β*-actin mRNA in the hypothalamus of rainbow trout fed one of the experimental diets; control (C), seaweed fly larvae (SWFL), black soldier fly larvae (BSFL), and reference commercial diet (Ref).

Gene	Diet				*p* -Value	
	C	SWFL	BSFL	Ref	Diet	Tank
CART	26.33 ± 20.55	28.52 ± 7.94	29.77 ± 67.81	31.88 ± 14.39	0.552	0.336
CRF	0.69 ± 0.28	0.36 ± 0.16	0.68 ± 0.57	0.27 ± 0.07	0.094^a^	0.213
MC4R	0.78 ± 0.04	0.81 ± 0.03	0.93 ± 0.16	0.89 ± 0.50	0.652^a^	0.945
NPY	1.40 ± 2.00	0.66 ± 0.99	1.78 ± 4.42	2.36 ± 31.32	0.275^b^	
POMCA1	1.41 ± 2.71	0.50 ± 0.70	1.12 ± 1.55	0.29 ± 0.49	0.322^b^	

*Note:* Data are shown as mean ± SD.

^a^ *p*-Values obtained from one-way ANOVA using log_10_ transformed data.

^b^ *p*-Values obtained from a non-parametric Kruskal–Wallis ANOVA and no nested model.

**Table 8:** Histological analysis of proximal and distal intestine of rainbow trout fed one of the experimental diets; control (C), seaweed fly larvae (SWFL), black soldier fly larvae (BSFL), and reference commercial diet (Ref).

Parameter	C	SWFL	BSFL	Ref	*p* -Value
*Proximal intestine*					
Villi length (µm)	463.42 ± 77.4	504.65 ± 101.8	451.99 ± 93.6	423.35 ± 87.9	0.493
Submucosa width (µm)	18.78 ± 4.19	16.56 ± 3.86	20.43 ± 5.75	16.58 ± 4.28	0.411
Lamina propria width (µm)	13.32 ± 1.72^a^	13.32 ± 1.52^a^	13.57 ± 2.74^a^	17.34 ± 2.15^b^	0.007
Goblet cell density (/100 µm)	5.59 ± 0.89	4.38 ± 2.77	3.90 ± 1.97	5.66 ± 1.84	0.342
Vacuolization score	1.00 ± 0.00	1.00 ± 0.00	1.00 ± 0.00	1.00 ± 0.00	—
*Distal intestine*					
Villi length (µm)	423.78 ± 128.6	429.12 ± 96.2	501.92 ± 201.2	433.89 ± 98.0	0.739
Submucosa width (µm)	21.40 ± 10.12	19.83 ± 5.89	16.53 ± 3.12	14.69 ± 3.63	0.792^c^
Lamina propria width (µm)	11.66 ± 2.64	14.72 ± 4.33	10.30 ± 1.84	12.44 ± 2.05	0.092
Goblet cell density (100 µm)	4.50 ± 2.71	5.47 ± 2.08	4.00 ± 0.97	5.76 ± 3.45	0.583
Vacuolization score	1.00 ± 0.00	1.00 ± 0.00	1.00 ± 0.00	1.00 ± 0.00	—

*Note*: Data are shown as mean ± SD (*n* = 6). Values within rows with different superscripts are significantly different (*p* < 0.05). Vacuolization scoring system adopted from Knudsen et al. [16]: 1 = large vacuoles occupy almost the entire apical part of the enterocytes, 2 = medium-sized vacuoles, 3 = small-sized vacuoles are near the apical membrane in most enterocytes, 4 = scattered small vacuoles are still present in some enterocytes, and 5 = no supranuclear vacuoles are present.

^c^ *p*-Values obtained from a non-parametric Kruskal–Wallis ANOVA.

**Table 9:** Plasma chemistry of rainbow trout fed one of the experimental diets; control (C), seaweed fly larvae (SWFL), black soldier fly larvae (BSFL), and reference commercial diet (Ref).

Parameter	Diet				*p* -Value	
	C	SWFL	BSFL	Ref	Diet	Tank
Hematocrit (%)	32.0 ± 7.9^a^	35.8 ± 4.3^ab^	32.9 ± 8.3^a^	38.7 ± 5.6^b^	<0.001^d^	0.020
Hemoglobin (g/dL)	6.19 ± 1.59^a^	7.18 ± 0.97^bc^	6.50 ± 1.29^ab^	8.02 ± 1.12^c^	<0.001	0.138
MCHC (g/dL)	19.8 ± 1.5	20.1 ± 1.5	20.1 ± 2.2	20.8 ± 1.1	0.125	0.182
Glucose (ng/mL)	6.16 ± 1.03	6.23 ± 1.37	7.02 ± 2.03	6.83 ± 1.42	0.196^e^	—
Cortisol (ng/mL)	7.41 ± 6.43^ab^	8.71 ± 8.85^a^	4.87 ± 5.15^b^	8.35 ± 8.00^a^	0.018^d^	<0.001
Ghrelin (pM)	22.9 ± 5.6	24.2 ± 13.1	34.2 ± 16.6	30.5 ± 23.3	0.352^e^	—

*Note*: Data are shown as mean ± SD (*n* = 24). Values within rows with different superscripts are significantly different (*p* < 0.05).

Abbreviation: MCHC, mean corpuscular hemoglobin concentration.

^d^ *p*-Values obtained using log_10_ transformed data.

^e^ *p*-Values obtained from a non-parametric Kruskal–Wallis ANOVA.

## Data Availability

The data used in this article are available upon request.

## Supporting Information

Supporting Information Supporting Information: Additional supporting information can be found online in the Supporting Information section. A detailed chemical composition of the three experimental diets can be found in the supplement (Table S1 and Table S2).
