# Supplementary material for: Seaweed Fly Larvae Cultivated on Macroalgae Side Streams: A Novel Marine Protein and Omega-3 Source for Rainbow Trout
Source: Aquac Nutr. 2024 Oct 7;2024:4221883. doi: 10.1155/2024/4221883 (PMC11473171; doi:10.1155/2024/4221883)
Supplement: Supporting Information — Additional supporting information can be found online in the Supporting Information section. A detailed chemical composition of the three experimental diets can be found in the supplement (Table S1 and Table S2). [file 4221883.f1.docx]

Detailed chemical composition of the three experimental diets (Table A1 and Table A2).

| Table A1  Amino acid profile of the experimental diets; control (C), seaweed fly larvae (SWFL) and black soldier fly larvae (BSFL) on dry matter basis (g/100 g DM). | | | |
| --- | --- | --- | --- |
|  | C | SWFL | BSFL |
| Indispensable amino acids |  |  |  |
| Arginine | 2.48 | 2.23 | 2.25 |
| Histidine | 0.99 | 1.01 | 0.96 |
| Isoleucine | 1.69 | 1.66 | 1.69 |
| Leucine | 3.20 | 3.02 | 3.09 |
| Lysine | 2.39 | 2.19 | 2.14 |
| Methionine | 1.60 | 1.59 | 1.63 |
| Phenylalanine | 2.06 | 2.05 | 2.03 |
| Threonine | 1.61 | 1.56 | 1.55 |
| Valine | 1.90 | 1.85 | 1.95 |
|  |  |  |  |
| Dispensable amino acids |  |  |  |
| Alanine | 1.99 | 2.04 | 1.99 |
| Aspartic acid | 3.58 | 3.37 | 3.49 |
| Cysteine | 0.61 | 0.55 | 0.56 |
| Glutamic acid | 9.62 | 9.42 | 9.11 |
| Glycine | 2.13 | 1.93 | 1.98 |
| Proline | 2.92 | 2.98 | 3.10 |
| Serine | 2.10 | 1.93 | 2.00 |
|  | | | |

| Table A2  Fatty acid composition (% of total fatty acids) of the experimental diets; control (C), seaweed fly larvae (SWFL) and black soldier fly larvae (BSFL). | | | |
| --- | --- | --- | --- |
|  | C | SWFL | BSFL |
| C 12:0 |  | 0.2 | 2.7 |
| C 14:0 | 2 | 2.3 | 2.3 |
| C 14:1 n-5 |  | 0.1 |  |
| C 15:0 | 0.3 | 0.3 | 0.3 |
| C 16:0 | 12.4 | 12.6 | 12.6 |
| C 16:1 n-7 | 2.7 | 4.4 | 2.6 |
| C 17:0 | 0.2 | 0.2 | 0.2 |
| C 17:1 n-7 |  |  |  |
| C 18:0 | 1.9 | 1.9 | 2.0 |
| C 18:1 | 40.4 | 39.5 | 39.1 |
| C 18:2 n-6 | 13.2 | 12.5 | 13.7 |
| C 18:3 n-3 | 5.5 | 5.4 | 5.3 |
| C 18:3 n-6 |  | 0.1 |  |
| C 18:4 n-3 | 1.2 | 1.2 | 1.1 |
| C 20:0 | 0.3 | 0.3 | 0.3 |
| C 20:1 n-9 | 1.7 | 1.2 | 1.3 |
| C 20:4 n-6 | 0.3 | 0.5 | 0.3 |
| C 20:4 n-3 | 0.3 | 0.3 | 0.2 |
| C 20:5 n-3 (EPA) | 4.2 | 4.1 | 3.7 |
| C 22:0 | 0.2 | 0.2 | 0.2 |
| C 22:1 | 1.4 | 0.8 | 0.9 |
| C 22:5 n-6 | 0.2 | 0.2 | 0.1 |
| C 22:5 n-3 | 0.4 | 0.4 | 0.4 |
| C 22:6 n-3 (DHA) | 6.6 | 6.2 | 6.0 |
| C 24:1 n-9 | 0.7 | 0.7 | 0.7 |
| Saturated fatty acids | 17.5 | 18.1 | 20.9 |
| Single unsaturated fatty acids | 47.0 | 46.9 | 44.6 |
| Polyunsaturated fatty acids | 32.4 | 31.3 | 31.4 |
| Omega n-6 fatty acids | 14.2 | 13.6 | 14.6 |
| Omega n-3 fatty acids | 18.2 | 17.7 | 16.8 |
| Omega n-6/n-3 ratio | 0.78 | 0.77 | 0.87 |
|  | | | |
